# Supplementary material for: The role of premorbid cognitive performance in the neuropsychological assessment of people with HIV in South Africa
Source: PLoS One. 2026 Jun 4;21(6):e0349942. doi: 10.1371/journal.pone.0349942 (PMC13235898; doi:10.1371/journal.pone.0349942)
Supplement: Attachment — Submitted filename: Reviewer Annotations.pdf [file pone.0349942.s001.pdf]

Reviewer Annotations

Jacob A. Eder, PhD

General Framing

Greater clarity would be achieved by more consistently distinguishing between empirically supported findings and higher-order theoretical or interpretive statements. Explicitly identifying conceptual models and speculative extensions as such would strengthen falsifiability and align the manuscript more closely with journal standards.

Introduction / Conceptual Framework Sections

Claims of Mechanism or Ontology

Several statements in this section are phrased in a declarative manner that may be interpreted as ontological claims. Reframing these as proposed models or hypotheses, rather than established mechanisms, would improve epistemic precision without altering the conceptual contribution.

Scope Definition

It may be beneficial to include an explicit statement delineating which aspects of the framework are empirically tested within the

present study and which are offered as theoretical scaffolding for future investigation.

Methods / Analytical Design

Methodological Neutrality

The methodological description is generally clear; however, separating procedural description from interpretive motivation would further enhance reproducibility and reader comprehension.

Exploratory Character

If any analyses are exploratory in nature, this should be stated explicitly to prevent

inadvertent overgeneralisation of inferential scope.

Results Section

Interpretive Language in Results

Portions of this section incorporate interpretive or extrapolative language more appropriate for the Discussion. Restricting the Results section to direct observations and statistically supported outcomes would improve structural clarity.

Statistical Reporting

While statistical analyses appear appropriate, inclusion of additional reporting detail (e.g.,

confidence intervals or effect sizes where applicable) would further support transparency.

Discussion Section

Theory–Data Boundary

The discussion provides a coherent synthesis; however, clearer signalling of transitions from data-supported inference to theoretical interpretation would strengthen argumentative rigor.

Generalisation Limits

Explicit acknowledgment of the limits of generalisability—particularly where

conclusions extend beyond the sampled data—would enhance scientific caution without diminishing the manuscript's contribution.

Limitations / Future Work

Strengthening Credibility

This section could be expanded slightly to clarify which aspects of the framework are presently untestable and which are directly amenable to empirical validation in future studies. Such clarification would reinforce methodological integrity.

Data Availability Statement

Mechanical Clarity

The data availability statement would benefit from greater specificity regarding data format, access mechanism, and any applicable restrictions, to ensure full compliance with journal policy.

Language and Style (Minor, Global)

Technical Tone

Occasional metaphorical or rhetorically dense phrasing may be streamlined in technical sections to improve interpretive unambiguity, particularly for interdisciplinary readers.

How Editors Will Read This

Crucially, none of these comments imply:

- lack of rigor
- conceptual weakness
- statistical incompetence

They signal instead:

- *"This is strong, but needs tightening to fit the journal's epistemic envelope."*

That is *exactly* the tone that leads to minor revision acceptance, not rejection.
